# Validity and Usability of Low-Cost Accelerometers for Internet-Based Self-Monitoring of Physical Activity in Patients With Chronic Obstructive Pulmonary Disease

**DOI:** 10.2196/ijmr.3056

**Published:** 2014-10-27

**Authors:** Martijn Vooijs, Laurence L Alpay, Jiska B Snoeck-Stroband, Thijs Beerthuizen, Petra C Siemonsma, Jannie J Abbink, Jacob K Sont, Ton A Rövekamp

**Affiliations:** ^1^Rijnlands Rehabilitation CenterDepartment of Cardiac and Pulmonary RehabilitationLeidenNetherlands; ^2^Leiden University Medical CenterDepartment of Medical Decision MakingLeidenNetherlands; ^3^TNOExpertise Center Life StyleLeidenNetherlands

**Keywords:** accelerometers, activity monitoring, chronic obstructive pulmonary disease, validity, usability

## Abstract

**Background:**

The importance of regular physical activity for patients with chronic obstructive pulmonary disease (COPD) is well-established. However, many patients do not meet the recommended daily amount. Accelerometers might provide patients with the information needed to increase physical activity in daily life.

**Objective:**

Our objective was to assess the validity and usability of low-cost Internet-connected accelerometers. Furthermore we explored patients’ preferences with regards to the presentation of and feedback on monitored physical activity.

**Methods:**

To assess concurrent validity we conducted a field validation study with patients who wore two low-cost accelerometers, Fitbit and Physical Activity Monitor (PAM), at the same time along with a sophisticated multisensor accelerometer (SenseWear Armband) for 48 hours. Data on energy expenditure assessed from registrations from the two low-cost accelerometers were compared to the well validated SenseWear Armband which served as a reference criterion. Usability was examined in a cross-over study with patients who, in succession, wore the Fitbit and the PAM for 7 consecutive days and filled out a 16 item questionnaire with regards to the use of the corresponding device

**Results:**

The agreement between energy expenditure (METs) from the SenseWear Armband with METs estimated by the Fitbit and PAM was good (*r*=.77) and moderate (*r*=.41), respectively. The regression model that was developed for the Fitbit explained 92% whereas the PAM-model could explain 89% of total variance in METs measured by the SenseWear. With regards to the usability, both the Fitbit and PAM were well rated on all items. There were no significant differences between the two devices.

**Conclusions:**

The low-cost Fitbit and PAM are valid and usable devices to measure physical activity in patients with COPD. These devices may be useful in long-term interventions aiming at increasing physical activity levels in these patients.

## Introduction

In patients with chronic obstructive pulmonary disease (COPD) being physically active is considered of great importance in adequate disease management. The importance of physical activity (PA) has been well-established in healthy people as it reduces the risk for chronic diseases, can favorably influence a broad range of physiological systems, and is associated with significant improvements in overall psychological well-being [1]. The American College of Sports Medicine (ACSM) therefore recommends adults to perform moderate-intensity aerobic (endurance) physical activity for a minimum of thirty minutes on at least five days a week [2], which is an internationally accepted standard. In patients with COPD, being physically active is of even greater importance as regular PA and an active lifestyle were shown to be positively associated with higher exercise capacity [3]. In addition to these benefits, patients with COPD performing some level of regular PA have a lower risk of both COPD-related hospital admissions and mortality [4,5], than patients that are less physically active.

Despite the importance of PA in patients with COPD, it seems difficult for the majority of COPD patients to meet the recommended amount of PA [6-8]. Compared to healthy controls, patients with COPD have significantly reduced duration, intensity, and counts (number of movements per day) of PA [9]. Initially, the reduced level of physical activity in COPD was attributed to decreased exercise capacity. However, several studies [10,11] showed that improved exercise capacity after eight to twelve weeks of pulmonary rehabilitation did not lead to a more active lifestyle, implying that enhanced function in patients with COPD may not translate directly into behavioral change. However, after six months of pulmonary rehabilitation increased activity levels were demonstrated [10], suggesting that a longer period of support is needed to achieve a change in physical activity behavior. While pulmonary rehabilitation programs are elaborate and expensive, other methods of support including support with the aid of the Internet could be considered to help patients with COPD to enhance their PA.

Pedometers or accelerometers are capable of measuring PA. While the former only measures steps, the latter can measure a wider range of activities. Internet-based self-monitoring of PA using accelerometers might be suitable to provide patients with the information and feedback needed to change PA behavior. Sophisticated accelerometers have proven valid in patients with COPD [12,13], and are recommended to assess patients’ PA for instance in the context of rehabilitation programs [14]. However, as these devices are costly and not intended for long-term Internet-based monitoring, alternatives need to be considered. For long-term Internet-based monitoring of PA at home, devices such as Fitbit or Physical Activity Monitor (PAM) are fairly inexpensive and commercially available.

In PRACTISS (Pulmonary RehAbilitation in COPD; Trial of sustained Self-management Support), a large randomized controlled trial, we are studying the one-year cost-effectiveness of an Internet-based self-management support system (PatientCoach) for patients with COPD. In this Internet-based self-management platform, ambulatory monitoring of physical activity plays an important part and in this context we evaluated accelerometers that met our predefined requirements for incorporation into PatientCoach.

To effectively support patients to self-manage their physical activities using a low-cost accelerometer for a longer period of time, certain prerequisites should be met. First of all, the device used should provide valid information about performed PA. Secondly, in order to wear a certain device for longer periods, it should be comfortable to wear, easy to use, intended users should be motivated to monitor their physical activity, and wearing the device should not arouse negative or unpleasant feelings.

We hypothesized that low-cost accelerometers meet these prerequisites. Therefore, we performed a validity study to assess the performance and a usability study to assess usability of such devices.

## Methods

### Patient Recruitment

Patients with COPD from the pulmonary rehabilitation department of the Rijnlands Rehabilitation Center in Leiden, the Netherlands, were prompted to take part in the studies. All patients contacted were involved in an outpatient, multidisciplinary pulmonary rehabilitation program between January and December 2012. In total, 25 patients participated: 9 in the validity study and 16 in the usability study. Patients participated in one of the studies within eight weeks from baseline pulmonary rehabilitation tests.

We collected patient characteristics such as age, gender, FEV_1_(L), post-bronchodilator FEV_1_(% predicted), exercise capacity measured by cardiopulmonary exercise testing (Watts) and peak VO_2_(ml/min) from the patient records. Patient characteristics are shown in Table 1.

**Table 1:** Patient characteristics.

Variable		Validity study	Usability study
		COPD-patients (n=9)	COPD-patients (n=16)
		mean (SD)	mean (SD)
Age in years		66.2 (4.4)	63.9 (10.0)
**Gender**			
	Male	5	6
	Female	4	10
BMI kg/m^2^		28.2 (5.4)	25.1 (5.7)
FEV_1_(L)		1.46 (0.74)	1.61 (0.63)
FEV_1_(%predicted)		51.1 (20.5)	57.4 (17.8)
FEV_1_/VC		0.39 (0.13)	0.46 (0.12)
MRC dyspnea (1-5)		3.0 (1.1)	2.3 (1.1)
**GOLD stage**			
	I	1	2
	II	4	9
	III	3	4
	IV	1	1
**GOLD patient group**			
	A	1	3
	B	1	6
	C	0	1
	D	7	6
**Smoking**			
	Smokers	2	5
	Nonsmokers	7	11
	Pack-years (packs per day × years as a smoker)	38.9 (15.6)	35.5 (11.8)

### The Devices

As we intended to incorporate the low-cost accelerometer into PatientCoach, the accelerometers had to meet our predefined requirements. First, data synchronization should be performed via wireless connection. Second, to enable PatientCoach to communicate with the external database, an Application Programming Interface (API) should be available. Finally, the cost of the accelerometer should not exceed US $150.

Two low-cost accelerometers (Table 2) met our predefined requirements and were hence evaluated, namely the Fitbit Ultra (Fitbit Inc, San Francisco, USA) and the Personal Activity Monitor AM300 (PAM BV Doorwerth, Netherlands).

Both the Fitbit and the PAM are three-axis accelerometers that measure motion patterns in three different planes. Besides the accelerometer, the Fitbit (FB) is also equipped with an altimeter to calculate the number of stairs climbed. The two low-cost accelerometers are both Internet-connected. This means that the data from these devices are uploaded to the Internet through wireless connection every time the device is in the vicinity of the included wireless receiver that is connected to a personal computer. The FB was worn in the right front trouser pocket, and the PAM on the waistband near the right hip as recommended by the manufacturers of the devices.

**Table 2:** Low-cost accelerometers.

Device	Technology	Output	Pricing
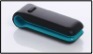 Fitbit Ultra	3-axis accelerometer Altimeter Wireless synchronization to Internet database	Energy expenditure Steps Stairs climbed	US $99
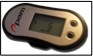 PAM AM300	3-axis accelerometer wireless synchronization to Internet database	PAM points	US $135

### Validity Study

In order to assess validity of both the FB and the PAM in daily living conditions we conducted a field validation study where we compared FB and PAM output with energy expenditure expressed as METs from the well-validated SenseWear Armband (SWA) from BodyMedia Inc. In order to assess the energy expenditure at home during 48 hours it was not an option to use indirect calorimetry as gold standard. Therefore, we used the validated SenseWear Armband as a criterion measure. In a one-hour standardized activity protocol, performed by COPD patients, the energy expenditure measured by the SWA previously showed a correlation of *r*=.76 (95% CI .54-.91) when compared to indirect calorimetry [12]. When compared with doubly labeled water, the SWA showed an ICC of .76 (95% CI .47-.90) on total energy expenditure over a 14-day period in women with COPD [13].

After a physiotherapist at the rehabilitation center had properly attached the devices, each patient wore the two low-cost accelerometers as well as the SWA simultaneously for 2 consecutive days at home during the daytime. Patients were instructed to re-attach the devices in the exact same position they were attached at the rehabilitation centre when they had to detach the devices, for instance when changing clothes or after bathing or sleeping. After 2 days, patients returned to the rehabilitation centre where the devices were collected for analysis.

### Usability Study

For the assessment of usability of two low-cost accelerometers we used a cross-over design study where COPD patients, who had never used either device before, were asked to wear the FB and PAM each for 7 consecutive days during the daytime. Participants were instructed to attach the devices to their waistband close to the hip (PAM) or trouser pocket (FB), immediately after waking up and to continue to wear the device until going to sleep. Block-randomization determined the order in which activity monitors were worn (PAM-FB or FB-PAM). The devices’ usability was measured by a self-developed 16-item usability questionnaire. After each 7-day period patients were asked to what extent they agreed with 16 statements regarding ease-of-use, usefulness, and acceptability of the corresponding accelerometer using a seven point Likert scale (1=disagree totally, 2=disagree strongly, 3=disagree slightly, 4=neutral, 5=agree slightly, 6=agree strongly, 7=agree totally). The questions were grouped in six domains which are presented in Table 3.

### Data Analysis

#### Validity Study

Data, which were stored by the devices, were downloaded to a personal computer. Data from the devices included steps (FB), stairs (FB), energy expenditure (FB), and PAM-score (PAM). Metabolic equivalents (METs) are used as a means of expressing the intensity and energy expenditure of activities. By convention one MET represents an energy expenditure of one kilocalorie per kilogram of body mass per hour. The PAM-score is an index representing the ratio of energy expended through physical activity to resting metabolism. MET values from the SWA were used as a reference standard for energy expenditure and were compared with energy expenditure (FB) and PAM-score.

Using linear regression analysis with step counts and calories (FB) and PAM-score and METs/3hr from the SWA as independent variable we estimated METs for FB and PAM. PA was expressed as mean METs per three-hour periods, since these time periods were found to provide sufficient detail for feedback on PA. In an additional linear regression model we included a dummy variable for each patient in order to adjust METs for the individual patient level. This allows the analysis of the agreement of changes in METs in individual patients between FB, PAM, and SWA, respectively. The agreement of mean METs/3hr between the Internet-based accelerometers and the gold standard was analyzed by the intra-class correlation coefficient (ICC) and Bland-Altman plots. Energy expenditure from FB and PAM was derived for every patient and then plotted in an identity plot against METs from the SWA. Subsequently, correlations (Pearson Correlation Coefficient) between these parameters were calculated. In order to investigate the total variance potentially explained by the devices, their output information was inserted into linear regression models to investigate the total variance (*R* ^*2*^) in METs explained by each device. Using linear regression modeling an algorithm to estimate METs was developed for FB and PAM, using the SWA as reference standard. Patient characteristics such as age, gender, FEV_1_(L), FEV_1_(% predicted), exercise capacity measured by cardiopulmonary exercise testing (Watts), and peak VO_2_(ml/min) were inserted into the model. The models were constructed using PAM-score from the PAM and steps and calories from the FB on a 3-hour basis. To correct for individual effects we constructed separate models which corrected for these effects.

#### Usability Study

For each domain of questions an average score was calculated, and differences between average domain scores for FB and PAM were compared using a paired *t* test. Furthermore, differences between males and females were investigated by calculating mean scores for women and men for each domain. Existing between-group differences were tested with an unpaired *t* test.

## Results

### Validity Study

Patient characteristics are shown in Table 1. Of the patients, 1 was excluded from the analysis due to technical problems, leaving 9 patients in the final analysis.

**Figure 1:**
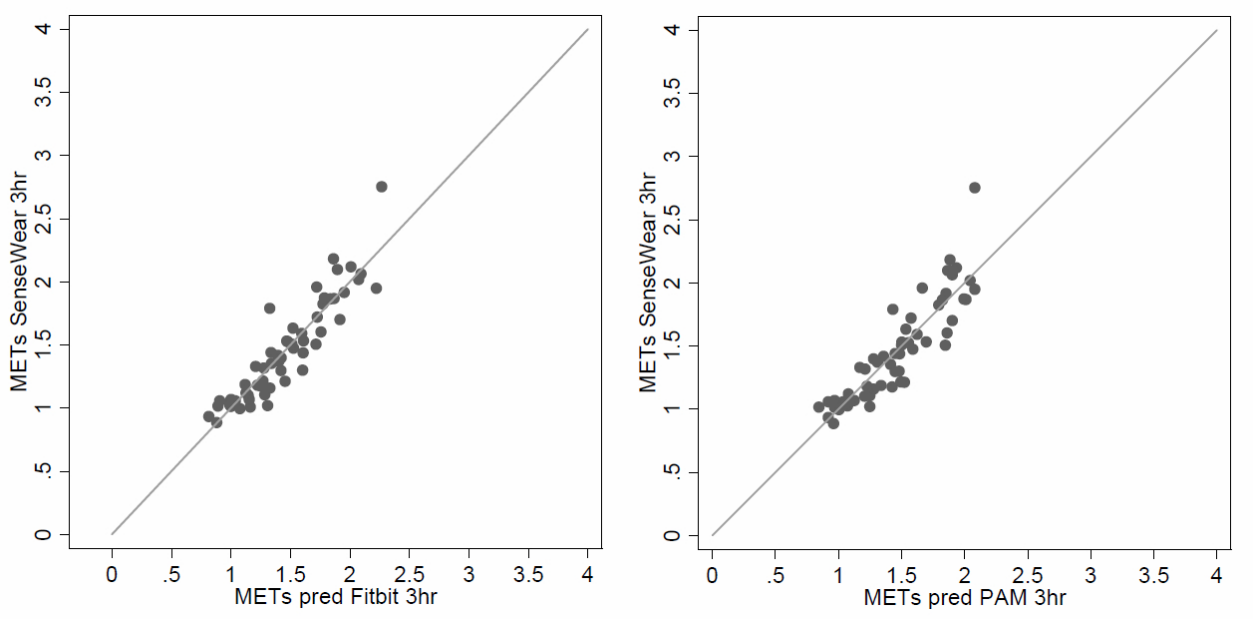
Identity plots of mean energy expenditure in METs/3 hours assessed by Fitbit and PAM compared to SenseWear.

Analysis showed that correlations per individual between METs (SWA) and energy expenditure (FB) ranged from 0.47 to 0.88 with a mean of 0.77 (95% CI 0.66-0.87) and correlations between METs (SWA) and PAM score from 0.18 and 0.61 with a mean of 0.41 (95% CI 0.30-0.53). Figure 1 shows the identity plots of the agreement in estimated mean METs/3hr between FB and PAM Internet accelerometers and SenseWear SWA, respectively. The regression model that was constructed to predict METs from FB output was able to explain 65% of total variance. After adding significant patient characteristics (length and sex) the explained variance improved to 67% and after correcting for patient effects to 85% (ICC= 0.92).

The model that was constructed to predict METs from PAM output was initially able to explain 53% of total variance which improved, after adding significant patient characteristics (length), to 70% and after correcting for patient effects to 81% (ICC= 0.89). The line of identity, indicating perfect agreement, has been drawn.

**Figure 2:**
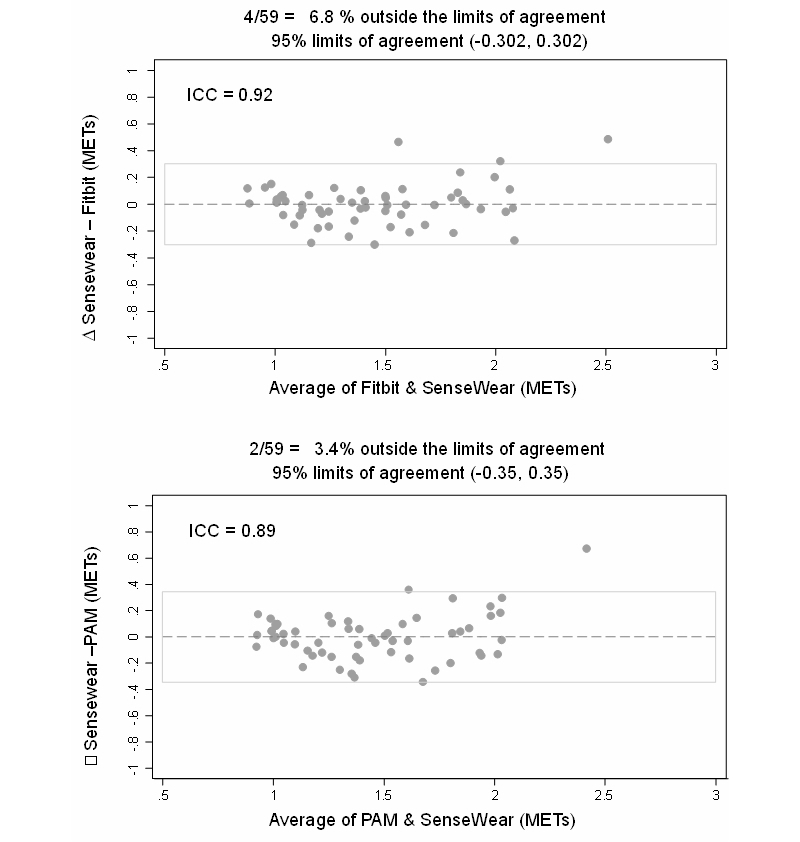
Bland Altman Plots for agreement in mean energy expenditure in METs/3 hours.

Figure 2 shows the Bland-Altman plots of the agreement in estimated mean METs/3hr between Fitbit and PAM Internet accelerometers and SenseWear Armband, respectively. There was good correlation between METs from SenseWear Armband, METs estimated by Fitbit (ICC=0.92), and PAM Internet accelerometers (ICC=0.89), respectively.

### Usability Study

Patient characteristics are listed in Table 1. Of the 19 patients who initially agreed to participate, 3 withdrew from the study. One patient found it psychologically too stressing to participate and two patients experienced problems installing the required software onto their computer, leaving 16 patients in the final analysis.

The different domains of usability and the results of the study are presented in Table 3. Overall, we found no statistically significant difference between the devices in any domain (*P*>.10) and overall usability score (*P*=.28). Additional between-group analyses revealed no significant differences between men and women for the different domains.

**Table 3:** Domains and findings from the usability questionnaire.

Domain	Number of questions	Mean score (score range 1-7)		Difference (*P* value)
		Fitbit	PAM	
Comfort in attaching and wearing the device (eg, easy to attach, comfortable to wear, wearing every day)	5	6.60	6.07	0.53 (*P*=.15)
Opinions towards wearing the device (eg, pleasant to wear, frightening to wear, frustrating to wear)	4	5.95	5.34	0.61 (*P*=.11)
Usefulness of activity monitoring (eg, useful to monitor activity, disadvantage to wear an activity monitor)	2	6.11	5.93	0.18 (*P*=.46)
Intention/willingness to monitor activity (eg, willingness to use an activity monitor/recommend to others)	3	5.53	5.17	0.36 (*P*=.23)
Technical aspects of the device (ie, it was easy to install the software)	1	4.85	4.95	0.10 (*P*=.82)
General appearance of the device (ie, device has an attractive appearance)	1	5.62	5.08	0.54 (*P*=.24)
Total usability score All questions	16	6.04	5.70	0.34 (*P*=.28)

## Discussion

### Principal Results

The present study shows that the Fitbit and PAM low-cost Internet-connected accelerometers have good validity and usability properties in order to monitor physical activity in patients with COPD. The Fitbit and PAM were both found to be valid when compared to the SenseWear Armband. Usability of the devices was well-rated with little difference between the Fitbit and the PAM. No negative or unpleasant feelings (frightening, frustrating) towards wearing the devices were reported during the usability study. In fact, the devices were found pleasant to wear and patients showed willingness to wear such a device for extended periods of time (>12 weeks), implying that they can be used outside the formal care settings. For instance, for supporting self-management of physical activity.

Our work shows that monitoring of physical activity using valid, user-friendly, and affordable devices is possible. Furthermore, patients with COPD show willingness to use these kinds of devices and are interested in monitoring their own physical activity.

### Limitations

Our studies inevitably have limitations. Firstly, patients included in the studies were participating or had already recently participated in a respiratory rehabilitation program, composing a convenience sample of patients that may have been (more) motivated to be physically active. This might indicate that our results do not necessarily apply to patients who were not involved in respiratory rehabilitation. However, as rehabilitation should be considered for all patients with chronic respiratory disease who have persistent symptoms, limited activity, and/or are unable to adjust to illness despite otherwise optimal medical management [15], our results might apply for COPD patients that have not yet been involved in rehabilitation, but are good candidates for doing so.

Secondly, the sample sizes for both studies are small and might not reflect COPD in general. However, we tried to use a representative sample of patients with COPD throughout both studies and as we took the frequency distribution of severity stages among patients with COPD in the Dutch population into account, it shows that in the usability study the frequency distribution of severity stages well represents the Dutch population: Gold I (13% vs 28%); Gold II (56% vs 54%); Gold III (25% vs 15%); and Gold IV (6% vs 3%). In the validity study the patients with very severe limitation are even a bit overrepresented (11% vs 3%) in the Dutch population.

Furthermore, we did not compare estimation of energy expenditure by the Fitbit and PAM with doubly-labeled water, as is recommended by the literature. Nevertheless, as the SenseWear was previously validated for estimating energy expenditure in patients with COPD [12,13,16-18], we found it justifiable to use it as a reference standard for comparison with new activity monitoring devices.

As we wanted to assess validity of the devices in real life conditions we did not control or directly influence the activities that were conducted by the patients. Variation in activity intensity was small in our sample, possibly limiting generalizability to activities with higher intensities. However, as our sample comprised patients from all four GOLD stages, the limited variation in exercise intensity might just reflect the actual activity patterns of this particular group.

The questionnaire used to measure usability has not been validated. However, it was based on the Unified Theory of Acceptance and Use of Technology (UTAUT) which was described in MIS Quarterly by Venkatesh et al [19], and the Post-Study System Usability Questionnaire (PSSUQ) by Lewis [20], where a seven-point Likert scale was used to assess acceptance and ease of use.

### Comparison to Related Work

Our studies focused on low-cost accelerometers for long-term self-management with regard to physical activity in persons with COPD. To our knowledge, the validity and usability of such devices have never been investigated in the targeted population. Previous studies have demonstrated validity of sophisticated activity monitoring devices [12,13,16-18], which, however, are not intended for long-term monitoring of PA and are fairly expensive. Low-cost pedometers have been successfully used in persons with COPD in the short-term (3 months) [21], and in children and adolescents [22]. The devices used in our studies provide information on both intensity and duration of activities rather than just reporting on step counts, thereby broadening the range of activities that can be conducted to increase physical activity.

Patients were positive regarding the usability of both devices. Both devices are quite small and discrete and were found comfortable to wear. Furthermore, patients showed interest in and willingness to monitor their PA. Finally, as there was no skin contact involved in wearing these devices, hygienic issues or skin reactions, which were found to be important issues in patients’ acceptance of wearable sensors [23], were not present.

As mentioned before, Internet-based activity monitoring is incorporated in PatientCoach, an interactive web-application to support COPD patients’ self-management. The evaluation of effectiveness of support by this system following a pulmonary rehabilitation program in COPD is currently ongoing in the PRACTISS trial (NTR 4009). In order to explore patients’ preferences with regards to the use of an activity monitor we organized a focus group at the Rijnlands Rehabilitation Centre with 5 COPD patients. Issues such as visual presentation of physical activity, feedback, and whether or not it was rewarding were addressed. Consistent with findings from van der Weegen et al [24], patients preferred simple and meaningful visualizations of activity data (active minutes per day). They also found it important to have an overview of activity results over several weeks or even months, and provided feedback should not be paternalistic.

### Implications

People with COPD can monitor their physical activity by using low cost Internet-based accelerometers. In the context of rehabilitation, this provides possibilities for COPD patients to monitor their PA between consultations, especially for gaining insight into any change and fluctuation. These can then be discussed with health care professionals (e.g. physician, physical therapist or specialized nurse) during a face-to-face consultation. These low-cost devices are also relevant to help patients monitor their PA after a rehabilitation treatment, knowing that PA tends to decrease during the post-rehabilitation period.

The results from our studies add knowledge that can be used for enhancing self-management of COPD patients, specifically regarding physical activity.

### Conclusions

Low cost Internet-based accelerometers can provide valid and useful estimates of within-person differences in metabolic equivalent level over three-hour periods in patients with COPD. These devices could provide information and feedback on longer-term PA in free-living conditions, and they are both user-friendly according to these mostly older patients. In the future, these devices may be useful in interventions aiming to increase physical activity levels by providing information and feedback on physical activity in patients with COPD.

## Multimedia Appendix 1

Usability and ease of use questionnaire.

## Abbreviations

ACSM: American College of Sports Medicine
API: application programming interface
BMI: body mass index
COPD: chronic obstructive pulmonary disease
FB: Fitbit
FEV1: Forced Expiration Volume in 1 second
GOLD: Global initiative for chronic Obstructive Lung Disease
ICC: intraclass correlation coefficient
MET: metabolic equivalent
MRC: Medical Research Council
NTR: Netherlands Trial Register
PA: physical activity
PAM: physical activity monitor
PRACTISS: Pulmonary RehAbilitation in COPD; TrIal of sustained Self-management Support
PSSUQ: post-study system usability questionnaire
SWA: Sensewear Armband
UTAUT: Unified Theory of Acceptance and Use of Technology

## References

[1] Blair SN, Wei M (2000). Sedentary habits, health, and function in older women and men. Am J Health Promot.

[2] Garber CE, Blissmer B, Deschenes MR, Franklin BA, Lamonte MJ, Lee IM, Nieman DC, Swain DP, American College of Sports Medicine (2011). American College of Sports Medicine position stand. Quantity and quality of exercise for developing and maintaining cardiorespiratory, musculoskeletal, and neuromotor fitness in apparently healthy adults: guidance for prescribing exercise. Med Sci Sports Exerc.

[3] Garcia-Aymerich J, Serra I, Gómez FP, Farrero E, Balcells E, Rodríguez DA, de Batlle J, Gimeno E, Donaire-Gonzalez D, Orozco-Levi M, Sauleda J, Gea J, Rodriguez-Roisin R, Roca J, Agustí AG, Antó JM, PhenotypeCourse of COPD Study Group (2009). Physical activity and clinical and functional status in COPD. Chest.

[4] Garcia-Aymerich J, Farrero E, Félez MA, Izquierdo J, Marrades RM, Antó JM, Estudi del Factors de Risc d'Agudització de la MPOC investigators (2003). Risk factors of readmission to hospital for a COPD exacerbation: a prospective study. Thorax.

[5] Watz H, Waschki B, Boehme C, Claussen M, Meyer T, Magnussen H (2008). Extrapulmonary effects of chronic obstructive pulmonary disease on physical activity: a cross-sectional study. Am J Respir Crit Care Med.

[6] Pitta F, Troosters T, Spruit MA, Probst VS, Decramer M, Gosselink R (2005). Characteristics of physical activities in daily life in chronic obstructive pulmonary disease. Am J Respir Crit Care Med.

[7] Watz H, Waschki B, Meyer T, Magnussen H (2009). Physical activity in patients with COPD. Eur Respir J.

[8] Garcia-Aymerich J, Félez MA, Escarrabill J, Marrades RM, Morera J, Elosua R, Antó JM (2004). Physical activity and its determinants in severe chronic obstructive pulmonary disease. Med Sci Sports Exerc.

[9] Vorrink SN, Kort HS, Troosters T, Lammers JW (2011). Level of daily physical activity in individuals with COPD compared with healthy controls. Respir Res.

[10] Pitta F, Troosters T, Probst VS, Langer D, Decramer M, Gosselink R (2008). Are patients with COPD more active after pulmonary rehabilitation?. Chest.

[11] Mador MJ, Patel AN, Nadler J (2011). Effects of pulmonary rehabilitation on activity levels in patients with chronic obstructive pulmonary disease. J Cardiopulm Rehabil Prev.

[12] Van Remoortel H, Raste Y, Louvaris Z, Giavedoni S, Burtin C, Langer D, Wilson F, Rabinovich R, Vogiatzis I, Hopkinson NS, Troosters T, PROactive consortium (2012). Validity of six activity monitors in chronic obstructive pulmonary disease: a comparison with indirect calorimetry. PLoS One.

[13] Farooqi N, Slinde F, Håglin L, Sandström T (2013). Validation of SenseWear Armband and ActiHeart monitors for assessments of daily energy expenditure in free-living women with chronic obstructive pulmonary disease. Physiol Rep.

[14] Glaab T, Vogelmeier C, Buhl R (2010). Outcome measures in chronic obstructive pulmonary disease (COPD): strengths and limitations. Respir Res.

[15] Spruit MA, Singh SJ, Garvey C, ZuWallack R, Nici L, Rochester C, Hill K, Holland AE, Lareau SC, Man WD, Pitta F, Sewell L, Raskin J, Bourbeau J, Crouch R, Franssen FM, Casaburi R, Vercoulen JH, Vogiatzis I, Gosselink R, Clini EM, Effing TW, Maltais F, van der Palen J, Troosters T, Janssen DJ, Collins E, Garcia-Aymerich J, Brooks D, Fahy BF, Puhan MA, Hoogendoorn M, Garrod R, Schols AM, Carlin B, Benzo R, Meek P, Morgan M, Rutten-van Mölken MP, Ries AL, Make B, Goldstein RS, Dowson CA, Brozek JL, Donner CF, Wouters EF, ATS/ERS Task Force on Pulmonary Rehabilitation (2013). An official American Thoracic Society/European Respiratory Society statement: key concepts and advances in pulmonary rehabilitation. Am J Respir Crit Care Med.

[16] Furlanetto KC, Bisca GW, Oldemberg N, Sant'anna TJ, Morakami FK, Camillo CA, Cavalheri V, Hernandes NA, Probst VS, Ramos EM, Brunetto AF, Pitta F (2010). Step counting and energy expenditure estimation in patients with chronic obstructive pulmonary disease and healthy elderly: accuracy of 2 motion sensors. Arch Phys Med Rehabil.

[17] Cavalheri V, Donária L, Ferreira T, Finatti M, Camillo CA, Cipulo Ramos EM, Pitta F (2011). Energy expenditure during daily activities as measured by two motion sensors in patients with COPD. Respir Med.

[18] Patel SA, Benzo RP, Slivka WA, Sciurba FC (2007). Activity monitoring and energy expenditure in COPD patients: a validation study. COPD.

[19] Venkatesh V, Morris MG, Davis GB, Davis FD (2003). MIS Quarterly.

[20] Lewis JR (1995). IBM computer usability satisfaction questionnaires: Psychometric evaluation and instructions for use. International Journal of Human-Computer Interaction.

[21] Moy ML, Weston NA, Wilson EJ, Hess ML, Richardson CR (2012). A pilot study of an Internet walking program and pedometer in COPD. Respir Med.

[22] Lubans DR, Morgan PJ, Tudor-Locke C (2009). A systematic review of studies using pedometers to promote physical activity among youth. Prev Med.

[23] Fensli R, Pedersen PE, Gundersen T, Hejlesen O (2008). Sensor acceptance model - measuring patient acceptance of wearable sensors. Methods Inf Med.

[24] van der Weegen S, Verwey R, Spreeuwenberg M, Tange H, van der Weijden T, de Witte L (2013). The development of a mobile monitoring and feedback tool to stimulate physical activity of people with a chronic disease in primary care: a user-centered design. JMIR Mhealth Uhealth.

