# Supplementary material for: Validity and Usability of Low-Cost Accelerometers for Internet-Based Self-Monitoring of Physical Activity in Patients With Chronic Obstructive Pulmonary Disease
Source: Interact J Med Res. 2014 Oct 27;3(4):e14. doi: 10.2196/ijmr.3056 (PMC4259960; doi:10.2196/ijmr.3056)
Supplement: Supplementary file 1 [file ijmr_v3i4e14_app1.pdf]

Usability and ease of use Questions:

1. I’m interested in measuring my daily physical activity.
2. It was easy to install the software on my computer.
3. It was easy to attach the activity monitor to my clothes.
4. The activity monitor was comfortable to wear.
5. The activity monitor has a nice appearance.
6. I found it reassuring to wear the activity monitor.
7. I found it frightening to wear the activity monitor.
8. I found it useful to use an activity monitor.
9. I found it frustrating to wear the activity monitor.
10. I wore the activity monitor every day.
11. I succeeded in wearing the activity monitor all day every dag.
12. I found it disadvantageous to wear the activity monitor every day.
13. I liked wearing the activity monitor.
14. I’m willing to wear an activity monitor in daily life.
15. I would be willing to wear the activity monitor for an extended period (>12 weeks).
16. I would recommend wearing an activity monitor to others.

Items were scored on a 7-point Likers scale
